# Photochemical synthesis of pyrano[2,3*-d*]pyrimidine scaffolds using photoexcited organic dye, Na_2_ eosin Y as direct hydrogen atom transfer (HAT) photocatalyst via visible light-mediated under air atmosphere

**DOI:** 10.1186/s13065-023-00912-7

**Published:** 2023-02-07

**Authors:** Farzaneh Mohamadpour

**Affiliations:** School of Engineering, Apadana Institute of Higher Education, Shiraz, Iran

**Keywords:** Na_2_ eosin Y photoexcited, Visible light mediated, Photochemical synthesis, Pyranopyrimidines

## Abstract

The Knoevenagel-Michael cyclocondensation of barbituric acid/1,3-dimethylbarbituric acid, malononitrile, and arylaldehyde derivatives was used to construct a multicomponent green tandem method for the metal-free synthesis of pyrano[2,3-*d*]pyrimidine scaffolds. At room temperature in aqueous ethanol, photo-excited state functions generated from Na_2_ eosin Y were employed as direct hydrogen atom transfer (HAT) catalysts by visible light mediated in the air atmosphere. This research looks towards expanding the use of a non-metallic organic dye that is both affordable and readily available. Because of its good yields, energy-effectiveness, high atom economy, time-saving qualities of the reaction, and operational simplicity, Na_2_ eosin Y is photochemically produced with the least amount of a catalyst. As a result, various ecological and sustainable chemical properties are met. Surprisingly, such cyclization may be carried out on a gram scale, indicating the reaction's potential industrial application.

**Supplementary Information:**

The online version contains supplementary material available at 10.1186/s13065-023-00912-7.

## Introduction

**Fig. 1:**
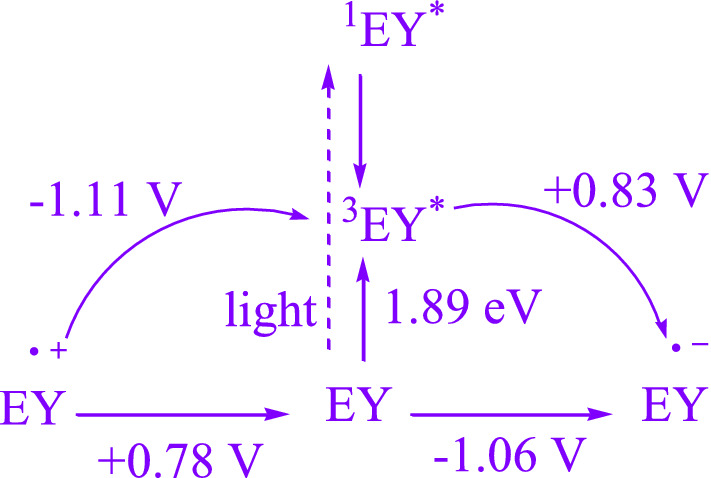
Eosin Y's oxidative and reductive quenching cycles, as well as their associated potentials [1]

EY is a readily available non-metallic organic dye which has recently found widespread use dueto its economic and ecological advantages over transition photocatalysts based on metal. At photoredox reactions mediated by eosin Y, the successfully oxidized/reduced intended substrates is often reliant on whether the substrates' prospective oxidability or reducibility is within the range of eosin Y (Fig. 1) [1–4].

**Fig. 2:**
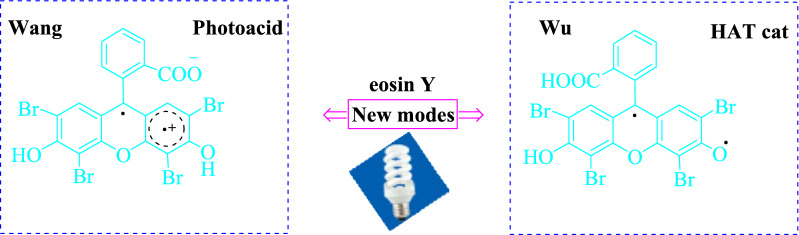
The photoinduced eosin Y being studied as a photoacid or HAT catalyst [1]

The spectrum of photochemical processes induced by eosin Y has been constrained by the aforementioned electrochemical requirements. In contrast to other natural dyes, eosin Y has singular phenol and xanthene moieties as well as strong acidic characteristics, resulting in four different formulations. There is substantial proof that anionic variants EY show photoactivity in the bulk of other photoreaction investigations, whereas the neutral forms are thought to be inactive and useless in potentially relevant synthesis methods [5, 6]. Wang et al. [7] and Wu et al. [8] were recently inspired by the characteristics of eosin Y to lead the discovery of novel photoinduced eosin Y activation states. The researchers discovered that induced modes generated from neutral eosin Y may act as direct HAT catalysts and photoacids for stimulating native Carbon Hydrogen bonds and glycals, respectively [1] (Fig. 2).

Hydrogen atom transfer (HAT) is a fundamental mechanism that may be involved in a variety of chemical, ecological, and biological systems. Direct HAT catalysis, assisted by quinone and benzophenone [9–11], was recently presented as a method to start C–H bonding in the presence of light.

Additionally, visible light radiation [12, 13] is a dependable method for green chemistry due to abundant energy resources, cheap price, and energy form in the synthesis of environmentally friendly organomolecules.

Pyranopyrimidines have been described with a variety of pharmacological properties as antihypertensive [14], cardiotonic [15], bronchiodilator [16], antibronchitic [17] and antitumor activities [18].

Numerous strategies are available [19–37]. Numerous instances occurred from these treatments. However, certain synthetic rules include limitations on the use of metal catalysts, severe reaction conditions, costly reagents, repetitive workup, low yield, prolonged reaction time, and environmental hazard.

Due to the aforementioned challenges and our concern for ecologically benign procedures, most scientists have been intrigued by the quest for easy, efficient, and environmentally safe methods that may enhance organic reactions under green conditions [38–40]. Considering the above concerns and our desire to build pyrano[2,3*-d*]pyrimidine scaffolds production, it is critical to investigate environmentally safe catalysts under green conditions for the correct synthesizing of the nitrogen heterocyclic complexes. This research establishes a novel function for the utilization of a non-metallic in aforementioned photochemical synthesizing process. There is proof that photoinduced states generated from Na_2_ eosin Y acts as a catalyst [41] for photochemical synthesizing through direct hydrogen atom transfer. This cyclocondensation at aqueous ethanol and room temperature and in an air environment is facilitated by visible light. This is a successful one-pot reaction carried out under very efficient, moderate, and simple conditions.

## Experimental

### General

All substances have their physical properties measured utilizing an Electro thermal 9100 equipment. Furthermore, the spectra were acquired utilizing nuclear magnetic resonance on a Bruker equipment (DRX-400 and DRX-300) with the solvent DMSO-d_6_.

Under white LED (18 W) irradiation, a combination of aryl aldehyde derivatives (**1**, 1.0 mmol), malononitrile (**2**, 1.0 mmol), and barbituric acid/1,3-dimethylbarbituric acid (**3**, 1.0 mmol) in an H_2_O/EtOH (2:1) (3 mL), was added Na_2_ eosin Y (1 mol %) and it was stirred, at room temperature. TLC was used to monitor the reaction's progression, using *n*-hexane/ethyl acetate (3:1) as the eluent. After the reaction occurs, the obtained material was screened and washed with water, and the crude solid was crystallized again from ethanol to get the pure substance with no further purifying. We wanted to see if we could scale up to the level that pharmaceutical process R&D wants, even if we were able to synthesize the above molecules using gram-scale techniques. 50 mmol of 2-methoxybenzaldehyde, malononitrile, and barbituric acid were combined in an experiment under standard conditions. The large-scale reaction went well and concluded in 12 min, with the product collected using standard filtration. The ^1^HNMR spectrum of this substance suggests that it is spectroscopically pure. After comparing spectroscopic data (^1^HNMR), the products were categorized. Spectral files some of the known products are offered Supporting Information file.

## Results and discussion

**Fig. 3:**
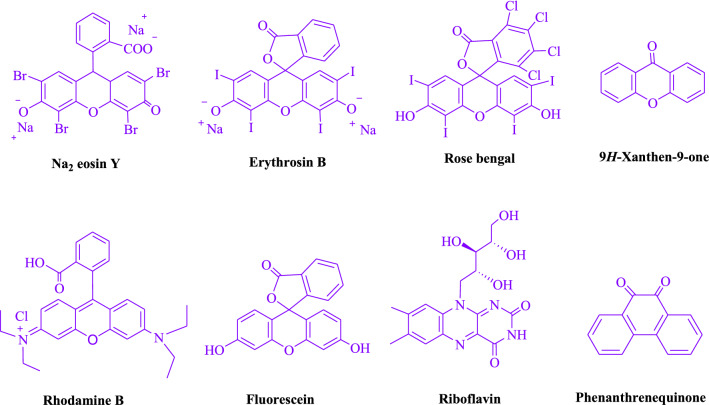
Photocatalysts tested in this study

**Fig. 4:**
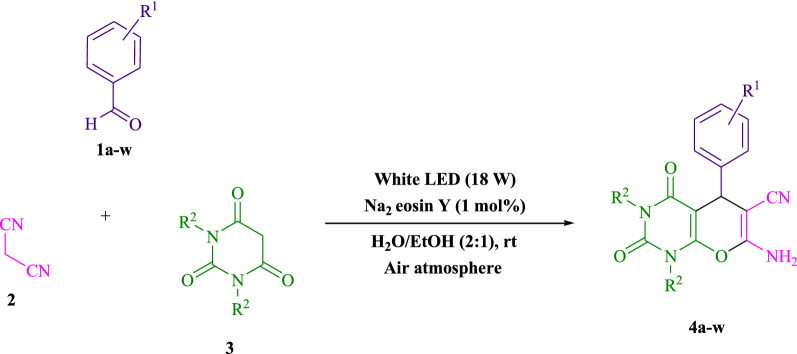
Synthesis of pyranopyrimidines

To begin, Table 1 summarizes the findings of an investigation into the reactivity of benzaldehyde, malononitrile, barbituric acid, EtOH/H_2_O (1:2) enhanced via irradiation at ambient temperature. With no photocatalyst, a 53% quantity of** 4a** was detected at room temperature for 25 min in EtOH/H_2_O (1:2). The process was facilitated by investigating a range of organophotocatalysts as rose bengal, erythrosin B, Na_2_ eosin Y, 9*H*-xanthen-9-one, rhodamine B, fluorescein, riboflavin and phenanthrenequinone (Fig. 3) under comparable conditions. The development of this phenomenon and the formation of the matching product **4a** were seen satisfactorily in yields ranging from 42 to 94% (Table 1). As per our results, Na_2_ eosin Y performed better than other photocatalysts in this process. By adding 1 mol% Na_2_ eosin Y, the yield was improved to 94% (Table 1, entry 4). Additionally, a poor product yield was observed in CH_2_Cl_2_, CH_3_CN, CHCl_3_, DMSO, DMF, toluene and THF (Table 2). By performing the reaction in H_2_O, EtOH, EtOAc, H_2_O/EtOH, solvent-free, MeOH, were increased the rate and yield of the reaction. A huge improvement was observed in H_2_O/EtOH (Table 2). The reaction went extremely well in H_2_O/EtOH (2:1), yielding 94% under similar circumstances (Table 2, entry 3). The yield was tested using a variety of illumination, showing that it increased somewhat in response to white LED. The finding demonstrates the critical nature of Na_2_ eosin Y and visible light for the product to develop effectively. Additionally, optimum conditions were found by changing the white LED irradiation intensities. Greatest results were obtained when white 18W LED irradiation was used. As shown in Fig. 4 and Table 3 this method is applicable to a variety of substrates.

**Table 1:** Photocatalyst optimization table


Entry	Photocatalyst	Solvent (3 mL)	Time (min)	Isolated Yields (%)
1	–	H_2_O/EtOH (2:1)	25	53
2	Na_2_ eosin Y (0.2 mol%)	H_2_O/EtOH (2:1)	10	71
3	Na_2_ eosin Y (0.5 mol%)	H_2_O/EtOH (2:1)	10	85
4	Na_2_ eosin Y (1 mol%)	H_2_O/EtOH (2:1)	10	94
5	Na_2_ eosin Y (1.5 mol%)	H_2_O/EtOH (2:1)	10	94
6	Erythrosin B (1 mol%)	H_2_O/EtOH (2:1)	10	42
7	Rose bengal (1 mol%)	H_2_O/EtOH (2:1)	10	63
8	9*H*-Xanthen-9-one (1 mol%)	H_2_O/EtOH (2:1)	10	52
9	Rhodamine B (1 mol%)	H_2_O/EtOH (2:1)	10	75
10	Fluorescein (1 mol%)	H_2_O/EtOH (2:1)	10	71
11	Riboflavin (1 mol%)	H_2_O/EtOH (2:1)	10	68
12	Phenanthrenequinone (1 mol%)	H_2_O/EtOH (2:1)	10	45

Reaction condition: malononitrile, benzaldehyde, barbituric acid, H_2_O/EtOH, White LED (18 W) with a variety of photocatalysts at room temperature

**Table 2:** Solvent and visible-light optimization table


Entry	Light Source	Solvent (3 mL)	Time (min)	Isolated Yields (%)
1	White light (18 W)	H_2_O/EtOH (1:1)	10	83
2	White light (18 W)	H_2_O/EtOH (1:2)	10	79
3	White light (18 W)	H_2_O/EtOH (2:1)	10	94
4	White light (18 W)	EtOH	10	61
5	White light (18 W)	H_2_O	10	74
6	White light (18 W)	MeOH	15	56
7	White light (18 W)	CH_2_Cl_2_	30	26
8	White light (18 W)	CH_3_CN	15	50
9	White light (18 W)	–	20	72
10	White light (18 W)	CHCl_3_	30	21
11	White light (18 W)	EtOAc	10	62
12	White light (18 W)	DMSO	25	47
13	White light (18 W)	DMF	40	25
14	White light (18 W)	Toluene	25	43
15	White light (18 W)	THF	40	28
16	White light (10 W)	H_2_O/EtOH (2:1)	10	75
17	White light (12 W)	H_2_O/EtOH (2:1)	10	82
18	White light (20 W)	H_2_O/EtOH (2:1)	10	94
19	–	H_2_O/EtOH (2:1)	25	< 5
20	Green light (18 W)	H_2_O/EtOH (2:1)	10	83
21	Blue light (18 W)	H_2_O/EtOH (2:1)	10	80

Reaction condition: malononitrile, benzaldehyde, barbituric acid, 1 mol% Na_2_ eosin Y

**Table 3:**
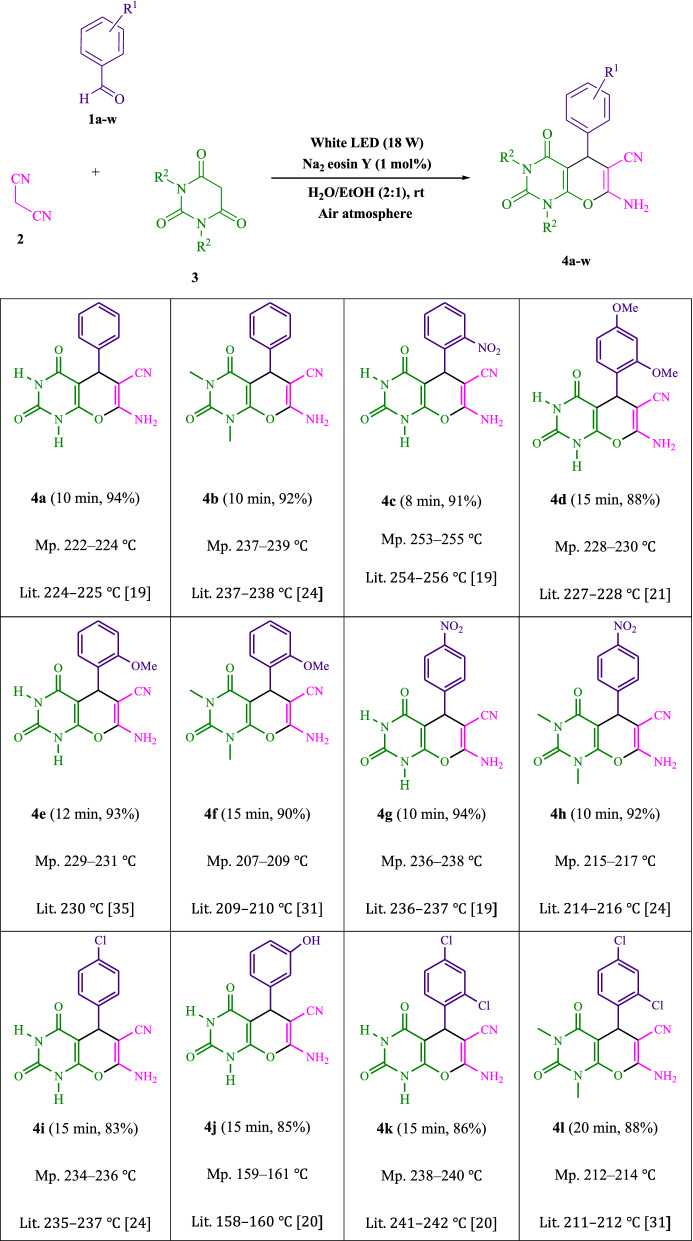

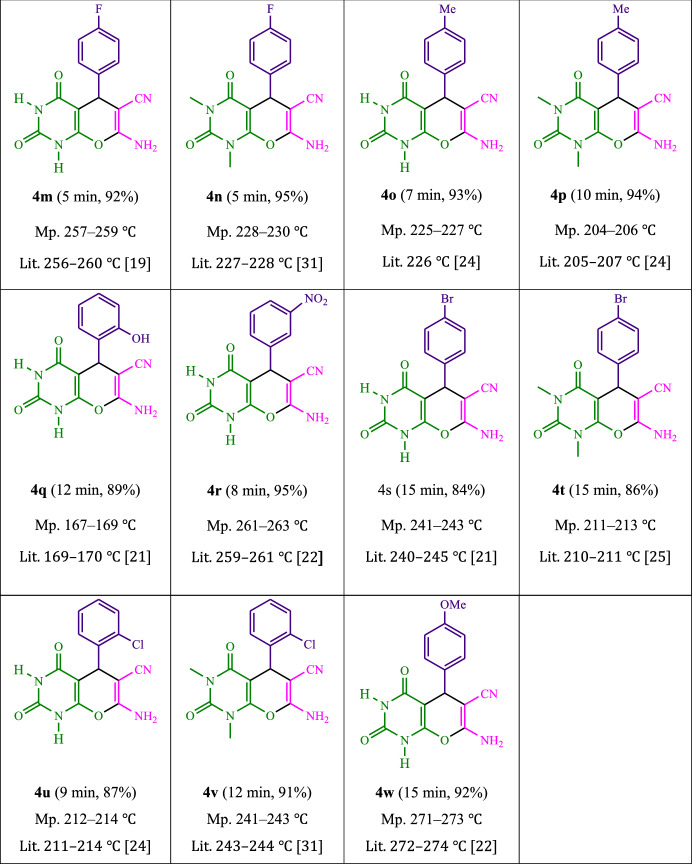
As a photocatalyst, photoexcited Na_2_ eosin Y was used for the synthesis of pyranopyrimidines

**Fig. 5:**
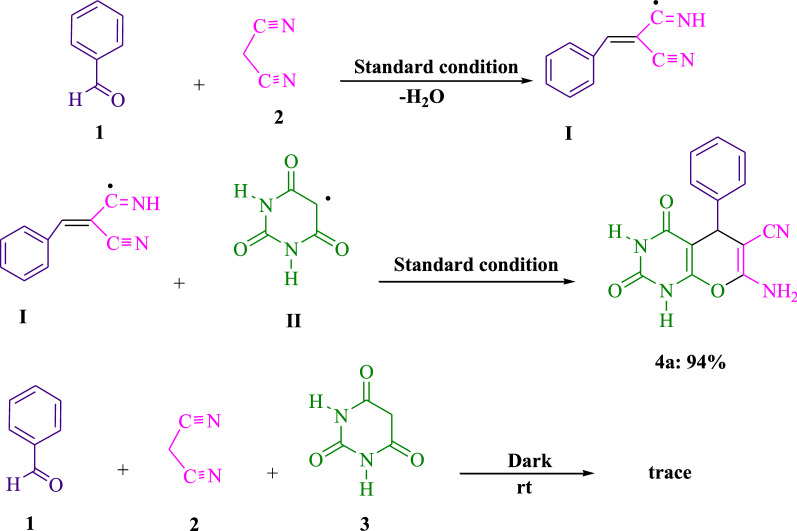
The reactions of benzaldehyde (**1**, 1 mmol), malononitrile (**2**, 1 mmol), barbituric acid (**3**, 1 mmol), and are crucial control tests for comprehending the reactions' mechanism

**Fig. 6:**
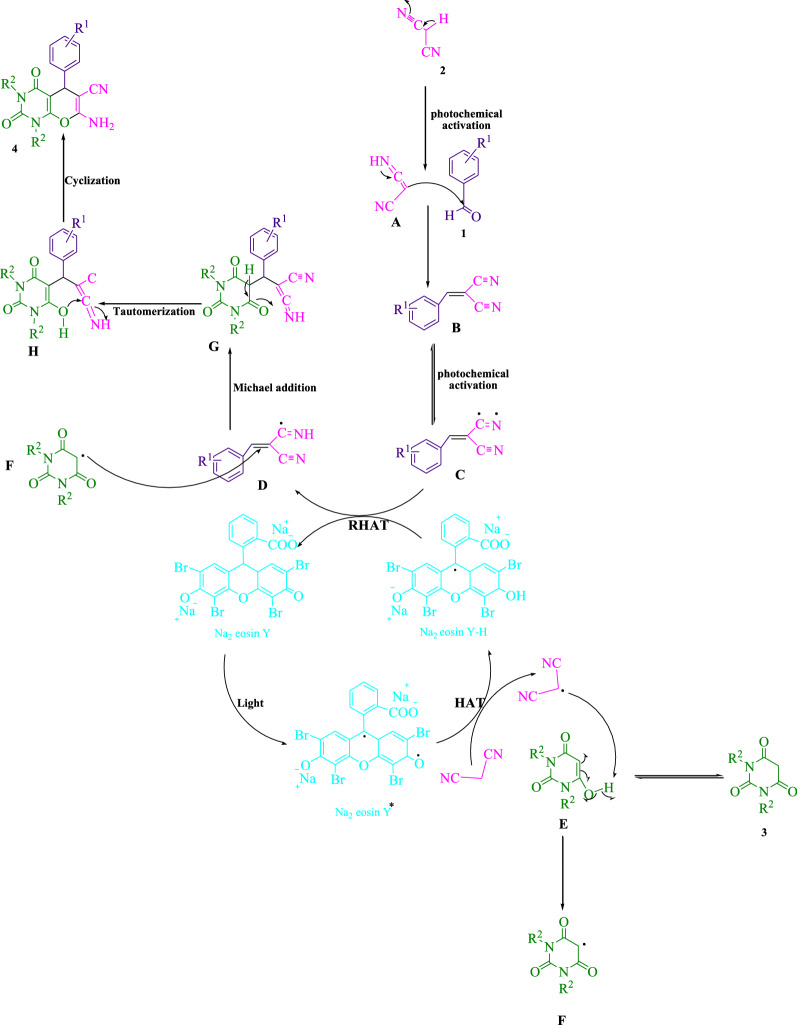
Recommended mechanistic path

Figure 5 illustrates the outcomes of the manage tests performed to decide the mechanism underlying this 3-component visible light-driven response. Within the first stage of the Knoevenagel-Michael cyclocondensation procedure, arylidenemalononitrile (**I**), which is produced, is condensed with (**II**). Under the following conditions (Na_2_ eosin Y in H_2_O/EtOH (2:1) and using white LED), malononitrile (**2**) and benzaldehyde (**1**) have been condensed to create arylidenemalononitrile (**I**) along with water removal. Then, the following reactions among the radical (**II**) and arylidenemalononitrile (**I**) led to the preferred product **4a** (94%). There was trace product **4a** created, even if the reaction was performed in general darkness. The effects of this experiment suggest that Fig. 6 offers a logical chemical pathway.

Figure 6 shows the suggested mechanism for synthesizing pyrano[2,3*-d*]pyrimidine scaffolds. With the use of visible light, malononitrile (**2**) is subjected tautomerization to give (**A**). Afterwards, (**A**) and aldehyde derivatives (**1**) react to form arylidenemalononitrile (**B**), undergoing an activation photochemically for the formation of a radical intermediate (**C**), in which visible light can partially affect with exerting extra energy to accelerate the reaction. As reported in previous studies [1, 4, 8], eosin Y-originated photoexcited modes can function as direct hydrogen atom transfer (HAT) catalysts for activating C-H bonds. The malononitrile radical is formed by the promotion of visible light triggered Na_2_ eosin Y* via a HAT procedure. Ground-state Na_2_ eosin Y and the intermediate (**D**) are regenerated by occurring reverse hydrogen atom transfer (RHAT) process between eosin Na_2_ Y-H and radical adduct **C**. Then, malononitrile radical extracts a hydrogen atom from (**E**) to produce intermediate (**F**). Subsequently, intermediate (**D**) and (**F**) coalesce to generate (**G**) as Michael acceptor, additionally undergoing tautomerization and intramolecular cyclization for the product formation (**4**).

Table 4 compares the catalytic capability of a variety of catalysts mentioned in this article. It may find a variety of uses, including the utilization of a little quantity of photocatalyst, a fast reaction time, and the absence of by-products when visible light irradiation is used. The atom-economic protocol is very successful at multigram scales and has significant industrial implications. These materials excel in terms of both efficiency and pureness. Table 4 also includes data on turnover number (TON) and turnover frequency (TOF). The higher the TON and TOF numerical values, the less catalyst is used and the greater the yield, and as the value rises, the catalyst becomes more effective.

**Table 4:** Comparative analysis of the catalytic properties of many of the catalysts mentioned in the text for producing **4a**

Entry	Catalyst	Conditions	Time/Yield (%)	TON	TOF	References
1	[DABCO](SO_3_H)_2_(Cl)_2_	H_2_O, Reflux	10 min/86	17.2	1.7	[19]
2	[DABCO](SO_3_H)_2_(HSO_2_)_2_	H_2_O, 90 °C	7 min/90	45	6.4	[19]
3	B(OH)_3_	THF/H_2_O, Reflux	125 min/81	8.1	0.06	[24]
4	DBA	EtOH/H_2_O, Reflux	58 min/94	4.7	0.08	[27]
5	Zn[(L)proline]_2_	EtOH, Reflux	60 min/85	5	0.08	[35]
6	theophylline	H_2_O/EtOH, 50 °C	10 min/86	8.6	0.8	[36]
7	β-CD	H_2_O, 80 °C	10 min/93	9.3	0.9	[37]
8	Na_2_ eosin Y	Visible light irradiation, H_2_O/EtOH (2:1), rt	10 min/94	94	9.4	This study

## Conclusion

In conclusion, the photoinduced states of Na_2_ eosin Y-derived act as a direct hydrogen atom transfer (HAT) catalyst was used for photochemically synthesizing pyrano[2,3*-d*]pyrimidine scaffolds through the three-condensation domino response of aryl aldehydes, malononitrile and barbituric acid/1,3-dimethylbarbituric acid in aqueous ethanol via visible light-mediated at room temperature. This study provides a green methodology for photochemically synthesizing with the least catalyst, producing good results, speeding. up the process, and achieving a high atom economy utilizing a non-metallic organic dye available commercially and at a low cost, Na_2_ eosin Y. This is a successful one-pot reaction that was carried out in a very efficient and straightforward manner.

## Supplementary Information

**Additional file 1: Fig. S1.** ^1^HNMR spectrum of compound (300 MHz, DMSO-d_6_) of **4d.** **Fig. S2.** 1HNMR spectrum of compound (300 MHz, DMSO-d6) of **4e**. **Fig. S3.** 1HNMR spectrum of compound (300 MHz, DMSO-d6) of **4m**. **Fig. S4.** 1HNMR spectrum of compound (300 MHz, DMSO-d6) of **4v**.

## Data Availability

All data generated or analyzed during this study are included in this published. article.

## References

[1] Yan DM, Chen JR, Xiao WJ (2019). New roles for photoexcited eosin Y in photochemical reactions. Angew Chem Int Ed.

[2] Wang X, Wang X, Xia C, Wu L (2019). Visible-light-promoted oxidative dehydrogenation of hydrazobenzenes and transfer hydrogenation of azobenzenes. Green Chem.

[3] Zhu J, Cui WC, Wang S, Yao ZJ (2018). Radical hydrosilylation of alkynes catalyzed by eosin Y and thiol under visible light irradiation. Org Lett.

[4] Chen MN, Di JQ, Li JM, Mo LP, Zhang ZH (2020). Eosin Y-catalyzed one-pot synthesis of spiro[4H-pyran-oxindole] under visible light irradiation. Tetrahedron.

[5] Hari DP, König B (2014). Synthetic applications of eosin Y in photoredox catalysis. Chem Commun.

[6] Majek M, Filace F, von Wangelin AJ (2014). On the mechanism of photocatalytic reactions with eosin Y. Beilstein J Org Chem.

[7] Zhao G, Wang T (2018). Stereoselective synthesis of 2-deoxyglycosides from glycals by visible-light-induced photoacid catalysis. Angew Chem Int Ed.

[8] Fan XZ, Rong JW, Wu HL, Zhou Q, Deng HP, Tan JD, Xue CW, Wu LZ, Tao HR, Wu J (2018). Eosin Y as a direct hydrogen-atom transfer photocatalyst for the functionalization of C−H bonds. Angew Chem Int Ed.

[9] Romero NA, Nicewicz DA (2016). Organic photoredox catalysis. Chem Rev.

[10] Ravelli D, Protti S, Fagnoni M (2016). Carbon–carbon bond forming reactions via photogenerated intermediates. Chem Rev.

[11] Capaldo L, Ravelli D (2017). Hydrogen atom transfer (HAT): a versatile strategy for substrate activation in photocatalyzed organic synthesis. Eur J Org Chem.

[12] Mohamadpour F (2021). Catalyst-free, visible light irradiation promoted synthesis of spiroacenaphthylenes and 1*H*-pyrazolo[1,2-*b*]phthalazine-5,10-diones in aqueous ethyl lactate. J Photochem Photobiol A.

[13] Mohamadpour F (2020). Visible light irradiation promoted catalyst-free and solvent-free synthesis of pyrano[2, 3-*d*]pyrimidine scaffolds at room temperature. J Saudi Chem Soc.

[14] Furuya S, Ohtaki T. Eur Pat Appl, EP 608565, 1994 Chem Abstr 1994; 121, 205395w

[15] Heber D, Heers C, Ravens U (1993). Positive inotropic activity of 5-amino-6-cyano-1, 3-dimethyl-1, 2, 3, 4-tetrahydropyrido [2, 3-d] pyrim idine-2, 4-dione in cardiac muscle from guinea-pig and man. Part 6: Compounds with positive inotropic activity. Pharmazie.

[16] Coates WJ. Eur Pat, 351058 Chem Abstr 1990; 113: 40711

[17] Sakuma Y, Hasegawa M, Kataoka K, Hoshina K, Yamazaki N, Kadota T, Yamaguchi H. WO 91/05785 PCT Int Appl 1989 Chem Abstr 1991; 115: 71646

[18] Broom AD, Shim JL, Anderson GL (1976). Pyrido[2, 3-d]pyrimidines. IV. Synthetic studies leading to various oxopyrido [2, 3-d] pyrimidines. J Org Chem.

[19] Seyyedi N, Shirini F, Safarpoor M, Langarudi N (2016). DABCO-based ionic liquids: green and recyclable catalysts for the synthesis of barbituric and thiobarbituric acid derivatives in aqueous media. RSC Adv.

[20] Bararjanian M, Balalaei S, Movassagh B, Amani AM (2009). One-pot synthesis of pyrano[2, 3-d]pyrimidinone derivatives catalyzed by L-proline in aqueous media. J Iran Chem Soc.

[21] Sheihhosseini E, Sattaei Mokhatari T, Faryabi M, Rafiepour A, Soltaninejad S (2016). Iron ore pellet, a natural and reusable catalyst for synthesis of pyrano[2,3-d]pyrimidine and dihydropyrano[c]chromene derivatives in aqueous media. Iran J Chem Chem Eng.

[22] Sadeghi B, Bouslik M, Shishehbore MR (2015). Nano-sawdust-OSO_3_H as a new, cheap and effective nanocatalyst for one-pot synthesis of pyrano[2, 3-d]pyrimidines. J Iran Chem Soc.

[23] Sabour B, Hassan Peyrovi M, Hajimohammadi M (2015). Al-HMS-20 catalyzed synthesis of pyrano[2,3-d]pyrimidines and pyrido[2,3-d]pyrimidines via three-component reaction. Res Chem Intermed.

[24] Khazaei A, Alavi Nik HA, Moosavi-Zare AR (2015). Water mediated domino knoevenagel-michael-cyclocondensation reaction of malononitrile, various aldehydes and barbituric acid derivatives using boric acid aqueous solution system compared with nano-titania sulfuric acid. J Chin Chem Soc.

[25] Maddila SN, Maddila S, van Zyl WE, Jonnalagadda SB (2015). Mn doped ZrO_2_ as a green, efficient and reusable heterogeneous catalyst for the multicomponent synthesis of pyrano[2,3-d]pyrimidine derivatives. RSC Adv.

[26] Maleki A, Jafari AA, Yousefi S (2017). Green cellulose-based nanocomposite catalyst: design and facile performance in aqueous synthesis of pyranopyrimidines and pyrazolopyranopyrimidines. Carbohydr Polym.

[27] Bhat AR, Shalla AH, Dongre RS (2016). Dibutylamine (DBA): a highly efficient catalyst for the synthesis of pyrano[2,3-d]pyrimidine derivatives in aqueous media. J Taibah Univ Sci.

[28] Mobinikhaledi A, Bodaghi Fard MA (2010). Tetrabutylammonium bromide in water as a green media for the synthesis of pyrano[2,3-d]pyrimidinone and tetrahydrobenzo[b]pyran derivatives. Acta Chim Slov.

[29] Zolfigol MA, Ayazi-Nasrabadi R, Baghery S (2016). The first urea-based ionic liquid-stabilized magnetic nanoparticles: an efficient catalyst for the synthesis of bis (indolyl) methanes and pyrano[2,3-d]pyrimidinone derivatives. Appl Organomet Chem.

[30] Azarifar D, Nejat-Yami R, Sameri F, Akrami Z (2012). Ultrasonic-promoted one-pot synthesis of 4H-chromenes, pyrano[2,3-d]pyrimidines, and 4H-pyrano[2,3-c]pyrazoles. Lett Org Chem.

[31] Khazaei A, Ranjbaran A, Abbasi F, Khazaei M, Moosavi-Zare AR (2015). Synthesis, characterization and application of ZnFe_2_O_4_ nanoparticles as a heterogeneous ditopic catalyst for the synthesis of pyrano[2,3-d]pyrimidines. RSC Adv.

[32] Devi I, Kumar BSD, Bhuyan PJ (2003). A novel three-component one-pot synthesis of pyrano[2,3-d]pyrimidines and pyrido[2,3-d]pyrimidines using microwave heating in the solid state. Tetrahedron Lett.

[33] Khurana JM, Vij K (2013). Nickel nanoparticles as semiheterogeneous catalyst for one-pot, three-component synthesis of 2-amino-4H-pyrans and pyran annulated heterocyclic moieties. Synth Commun.

[34] Bodaghifard MA, Solimannejad M, Asadbegi S, Dolatabadifarahani S (2016). Mild and green synthesis of tetrahydrobenzopyran, pyranopyrimidinone and polyhydroquinoline derivatives and DFT study on product structures. Res Chem Intermed.

[35] Heravi MM, Ghods A, Bakhtiari K, Derikvand F (2010). Zn[(L)proline]_2_: an efficient catalyst for the synthesis of biologically active pyrano[2,3-d]pyrimidine derivatives. Synth Commun.

[36] Mohamadpour F (2021). Synthesis of pyran-annulated heterocyclic systems catalyzed by theophylline as a green and bio-based catalyst. Polycycl Aromat Compd.

[37] Mohamadpour F (2022). Supramolecular β-cyclodextrin as a Biodegradable and Reusable Catalyst Promoted Environmentally Friendly Synthesis of Pyrano[2,3-*d*]pyrimidine Scaffolds via Tandem Knoevenagel–Michael–Cyclocondensation Reaction in Aqueous Media. Polycycl Aromat Compd.

[38] Mohamadpour F (2021). Photoexcited Na_2_ eosin Y as direct hydrogen atom transfer (HAT) photocatalyst promoted photochemical metal-free synthesis of tetrahydrobenzo[*b*]pyran scaffolds via visible light-mediated under air atmosphere. J Taiwan Inst Chem Eng.

[39] Mohamadpour F (2021). New role for photoexcited organic dye, Na_2_ eosin Y via the direct hydrogen atom transfer (HAT) process in photochemical visible-light-induced synthesis of spiroacenaphthylenes and 1*H*-pyrazolo[1,2-*b*]phthalazine-5,10-diones under air atmosphere. Dyes Pigm.

[40] Mohamadpour F (2022). The development of imin-based tandem Michael-Mannich cyclocondensation through a single-electron transfer (SET)/energy transfer (EnT) pathway in the use of methylene blue (MB^+^) as a photo-redox catalyst. RSC Adv.

[41] Mohamadpour F (2022). New role for photoexcited Na_2_ Eosin Y via the direct hydrogen atom transfer process in photochemical visible-light-induced synthesis of 2-Amino-4*H*-chromene scaffolds under air atmosphere. Front Chem.

