# Supplementary material for: Photochemical synthesis of pyrano[2,3-d]pyrimidine scaffolds using photoexcited organic dye, Na2 eosin Y as direct hydrogen atom transfer (HAT) photocatalyst via visible light-mediated under air atmosphere
Source: BMC Chem. 2023 Feb 7;17(1):2. doi: 10.1186/s13065-023-00912-7 (PMC9906854; doi:10.1186/s13065-023-00912-7)
Supplement: Supplementary file 1 — Additional file 1: Fig. S1. 1HNMR spectrum of compound (300 MHz, DMSO-d6) of 4d. Fig. S2. 1HNMR spectrum of compound (300 MHz, DMSO-d6) of 4e. Fig. S3. 1HNMR spectrum of compound (300 MHz, DMSO-d6) of 4m. Fig. S4. 1HNMR spectrum of compound (300 MHz, DMSO-d6) of 4v. [file 13065_2023_912_MOESM1_ESM.pdf]

Supporting Information

Photochemical synthesis of pyrano[2,3-*d*]pyrimidine scaffolds using photoexcited organic dye, Na_2 eosin Y as direct hydrogen atom transfer (HAT) photocatalyst *via* visible light-mediated under air atmosphere

Farzaneh Mohamadpour *

School of Engineering, Apadana Institute of Higher Education, Shiraz, Iran

* *Corresponding author. mohamadpour.f.7@gmail.com*

7-Amino-5-(2,4-dimethoxyphenyl)-2,4-dioxo-2,3,4,5-tetrahydro-1H-pyrano[2,3-d]-pyrimidine-6-carbonitrile (4d)

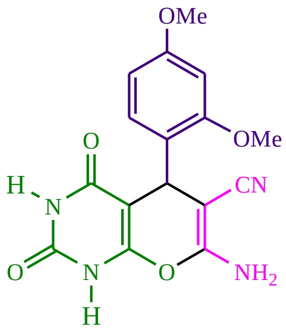

Yield: 88%; M.p. 228-230 °C; ^{1}HNMR (300 MHz, DMSO-d_6): 3.71 (3H, s, OCH_3), 3.76 (3H, s, OCH_3), 4.56 (1H, s, CHAr), 7.13-7.53 (5H, m, ArH & NH_2), 10.04 (1H, s, NH), 11.29 (1H, s, NH).

7-Amino-5-(2-methoxyphenyl)-2,4-dioxo-2,3,4,5-tetrahydro-1H-pyrano[2,3-d]-pyrimidine-6-carbonitrile (4e)

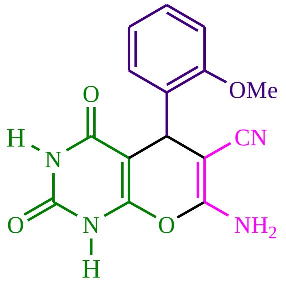

Yield: 93%; M.p. 229-231 °C; ^{1}HNMR (300 MHz, DMSO-d_6): 3.79 (3H, s, OCH_3), 4.49 (1H, s, CHAr), 7.46-7.78 (4H, m, ArH & NH_2), 8.18 (2H, t, J = 8.0 Hz, ArH), 10.58 (1H, s, NH), 11.36 (1H, s, NH).

7-Amino-5-(4-fluorophenyl)-2,4-dioxo-2,3,4,5-tetrahydro-1H-pyrano[2,3-d]-pyrimidine-6-carbonitrile (4m)

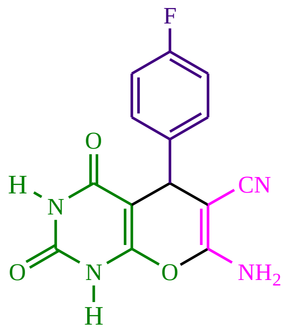

Yield: 92%; M.p. 257-259 °C; ^{1}HNMR (300 MHz, DMSO-d_6): 4.28 (1H, s, CHAr), 7.18-8.62 (6H, m, ArH & NH_2), 10.73 (1H, s, NH), 11.83 (1H, s, NH).

7-Amino-5-(2-chlorophenyl)-1,3-dimethyl-2,4-dioxo-2,3,4,5-tetrahydro-1H-pyrano[2,3-d]-pyrimidine-6-carbonitrile (4v)

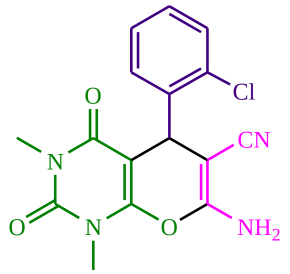

Yield: 91%; M.p. 241-243 °C; ^{1}HNMR (400 MHz, DMSO-d_6): 3.31 (3H, s, NCH_3), 3.36 (3H, s, NCH_3), 4.51 (1H, s, CHAr), 7.53-7.59 (4H, m, ArH & NH_2), 8.18 (2H, t, J = 6.8 Hz, ArH).

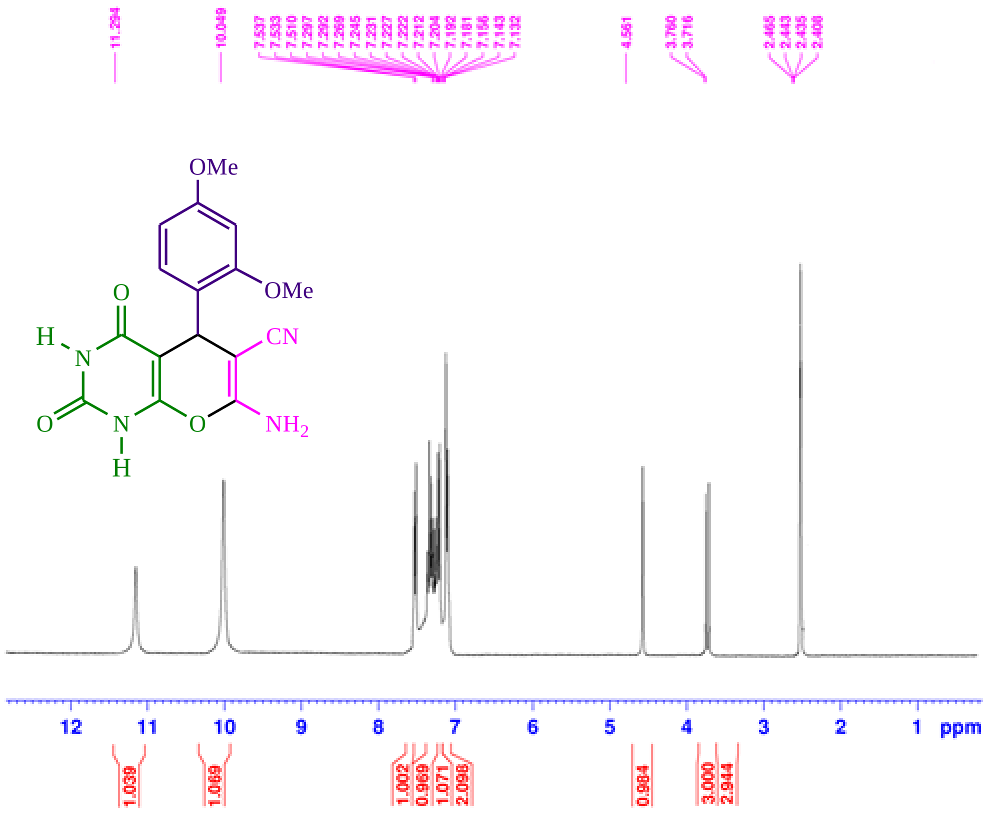

Fig. S1.^{1}HNMR Spectrum of compound (300 MHz, DMSO-d_6) of **4d**

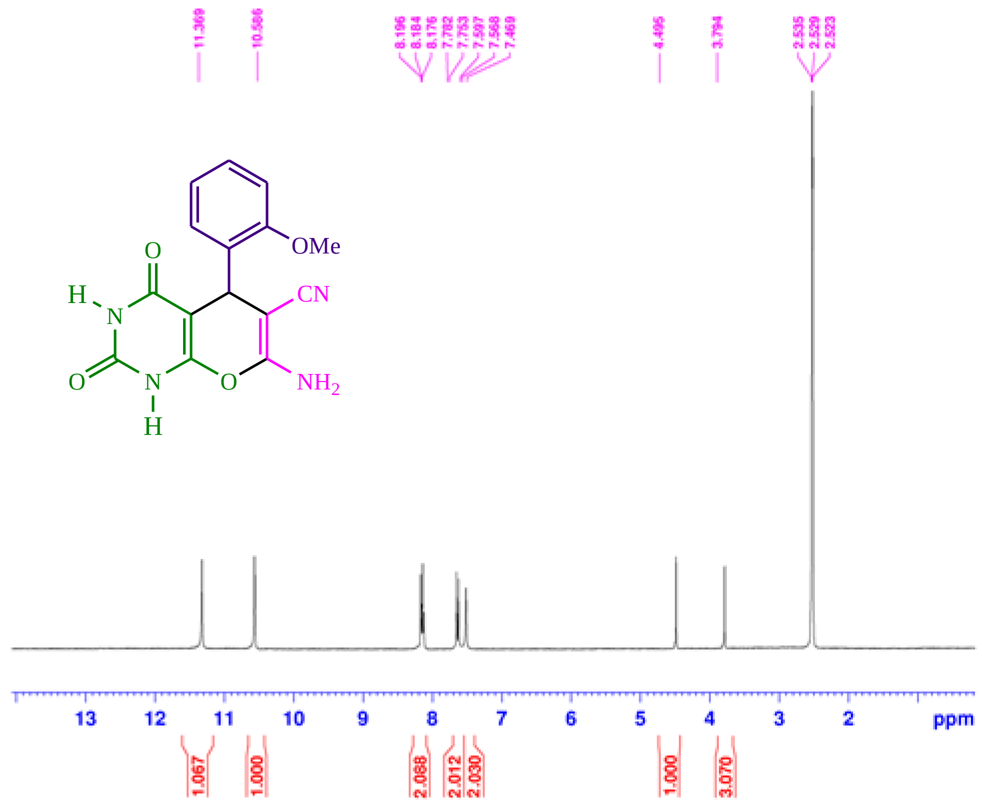

Fig. S2. ^{1}HNMR Spectrum of compound (300 MHz, DMSO-d_6) of **4e**

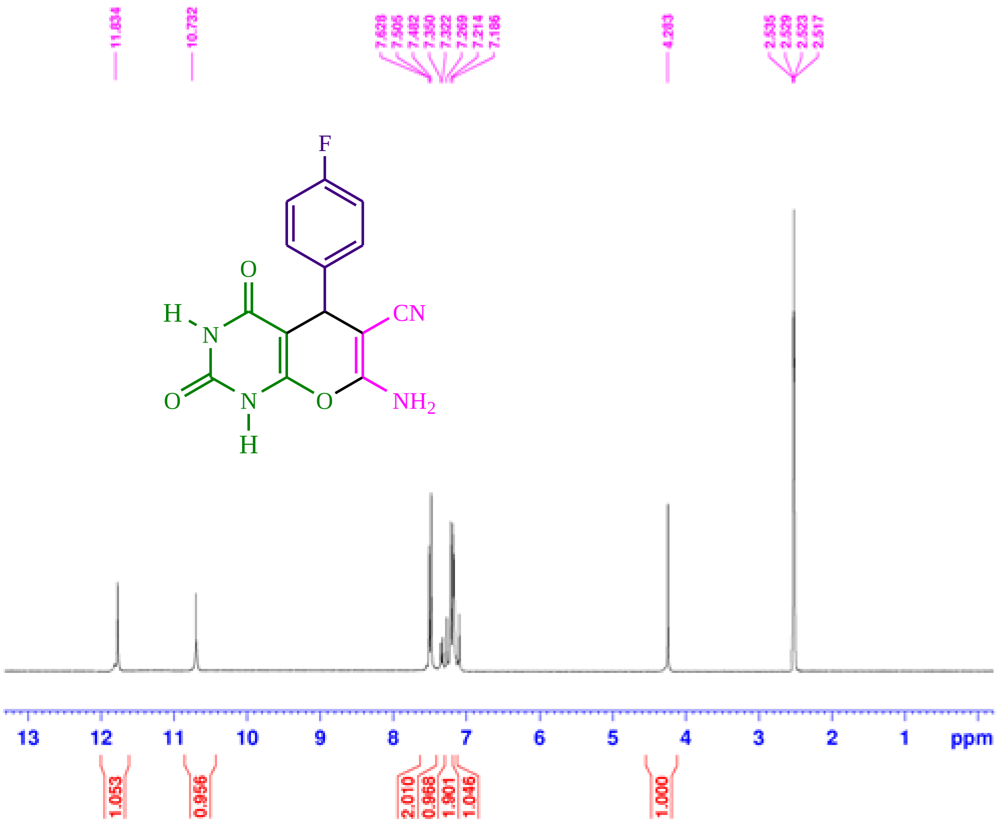

Fig. S3. ^{1}HNMR Spectrum of compound (300 MHz, DMSO-d$_6$) of **4m**

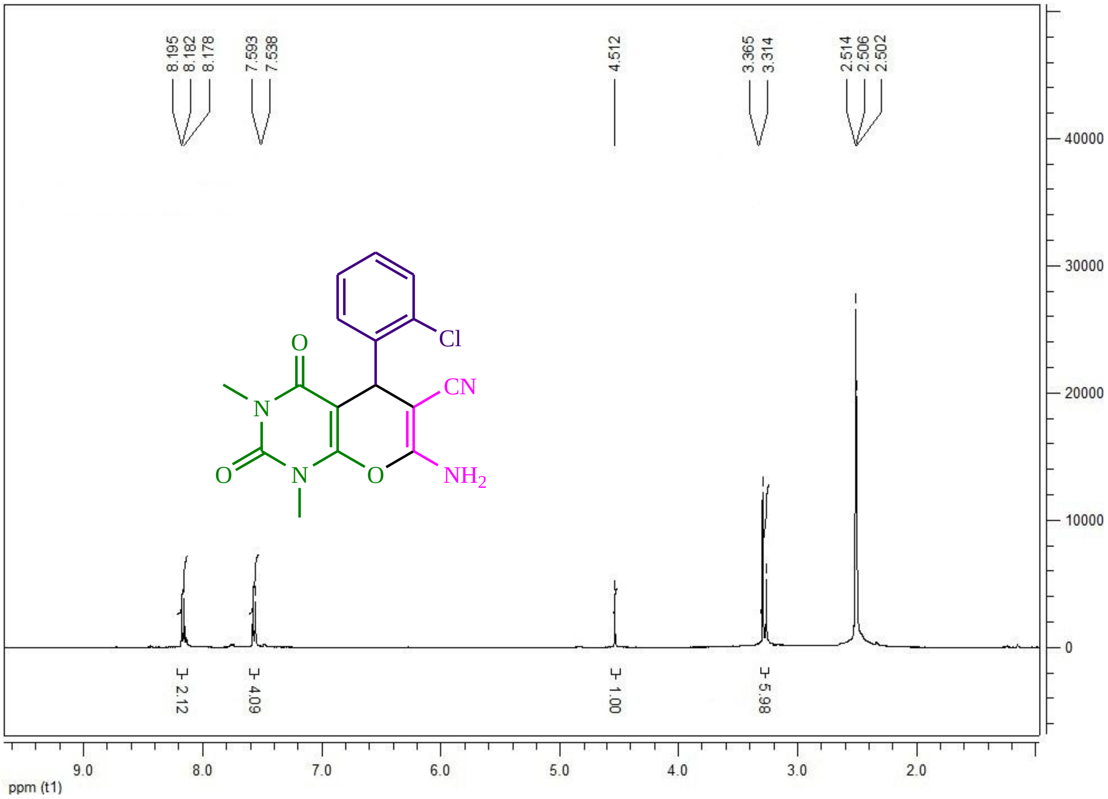

Fig. S4.^{1}HNMR Spectrum of compound (400 MHz, DMSO-d_6) of **4v**
